# A Holistic Ecosystem Model to Diversify the Physician Workforce and Enhance Health

**DOI:** 10.1177/24731242251371526

**Published:** 2025-08-25

**Authors:** Erik J. Porfeli, Sunny Nakae, Leila Amiri, Leila E. Harrison, Will Ross

**Affiliations:** ^1^ Department of Human Sciences, The Ohio State University, Columbus, Ohio, USA.; ^2^ California University of Science and Medicine, Colton, California, USA.; ^3^ The Robert Larner College of Medicine at the University of Vermont, Burlington, Vermont, USA.; ^4^ Washington State University Elson S. Floyd College of Medicine, Spokane, Washington, USA.; ^5^ Alumni Endowed Professor of Medicine, Division of Nephrology, Washington University School of Medicine, St. Louis, Missouri, USA.

**Keywords:** holistic review, workforce development, health disparities

## Abstract

**Importance:**

The U.S. medical education system attracts and trains the next generation of physicians to advance the health care needs of a growing and increasingly diverse nation. This system can be credited for supplying a physician workforce achieving remarkable growth and innovation, yielding one of the world’s most technologically advanced health care systems on the planet. This system, unfortunately, also contributes to educational, workforce, and health disparities.

**Observations:**

The successes and challenges of the medical education and health care system align with broader economic, health, and educational patterns in the United States. An ecological model can be employed to unite a network of partners spanning four developmental stages to support a greater diversity of students for and from underrepresented communities to enter the physician workforce, enjoy the rewards granted by a career in medicine, and enact needed changes to eliminate health, economic, and educational disparities.

**Conclusions and Relevance:**

Comprehensive and ecologically attuned pathways to the physician workforce could be especially beneficial to states and communities suffering from the looming high school enrollment cliff, outflows of residents to other states, challenges in recruiting and retaining physicians, and significant educational and health disparities. The ecosystem model spurs significant changes in how we think about the developmental pathways to the physician workforce and how we may mobilize resources to promote progress and ease transitions, especially for underrepresented students who face many fewer opportunities and many more challenges along their journey.

## Introduction

An analysis of the 2021 World Index of Healthcare Innovation found that the U.S. is a global leader in scientific advancement.^1^ The remarkable strength of our medical education and health care system is, however, diminished by its inequitable practices and outcomes. The US spent $4.3 trillion on health care in 2021, more than 18% of the country’s Gross Domestic Product.^2^ Yet, the U.S. pales in comparison with health outcomes of other countries within the Organization for Economic Co-operation and Development (OECD), ranking 34th in infant mortality, 32nd in total life expectancy, 32nd in suicide rates, and 38th in total obesity rates.^1^ These poor health outcomes are disproportionately borne by impoverished and minoritized populations. Abundant research documented persistent disparities among racial and ethnic minorities, who receive poorer health care across a range of illnesses and health care services even when access-related factors are taken into consideration.^3^ While the U.S. leads the world in many aspects of specialized medical care, much more innovation is needed to meet the health care needs of an increasingly diverse nation.^4^

The forces that play into health disparities translate into similar disparate patterns in medical education. According to a study by Lett et al.,^5^ the percentage of underrepresentation medical students has not changed significantly since 2009 when the Liaison Committee of Medical Education instituted its new diversity accreditation guidelines. In a review of enrollees in medical schools from 1978 through 2019,^6^ there were only modest changes in the percentages of Black women and men, which naturally translates into the same pattern in practicing physicians.

The U.S. medical education system struggles to matriculate underrepresented students partly because this aspect of the qualified applicant pool is far too small. Medical education further constrains this segment of the pool by placing a strong emphasis on academic metrics^7^ and special experiences (e.g., volunteer activities) that favor more economically advantaged applicants with the resources to achieve higher metrics and pursue unpaid experiences. Medical education is, therefore, both a producer and a product of these educational, health, and economic disparities reflected in the broader U.S. educational and health care systems.^8,9^

Recognizing the potential of medical education to mitigate educational, workforce, and health disparities, the Association of American Medical Colleges established an initiative promoting a holistic review process for medical school admissions. Holistic review refers to mission-aligned admissions or selection processes that take into consideration applicants’ experiences, attributes, and academic metrics as well as the value an applicant would contribute to learning, practice, and teaching.^10,11^ Holistic review empowers admissions committees to consider the “whole” applicant rather than disproportionately focusing on any one characteristic. Applicant selection criteria are broad, clearly linked to the school’s mission and goals, and promote numerous aspects of diversity as essential to excellence.

Subsequent research showed that the use of mission-driven holistic review increased the diversity of medical school and residency applicants who were interviewed and granted offers.^11,12^ These kinds of promising results supported the widespread adoption of the holistic review model in the United States and abroad. A recent survey found that 91% of U.S. medical schools reported using holistic review in student admissions.^12^ This model also demonstrated promise in residency selection.^13^ Despite extensive and commendable holistic review and pathway programs, the diversity of medical students, residents, and faculty from underrepresented groups and impoverished backgrounds has largely been unchanged for decades.^14,15^ A series of legal rulings over the past several years has further constrained aspects of the holistic review model to achieve its promise.^16,17^ Enrollment of underrepresented students has declined by over 30% in public medical schools in states where affirmative action was outlawed.^18^ A nationwide turning point occurred in June 2023, when the US Supreme Court ruled that affirmative action was unconstitutional.^19^ These mounting nationwide legal changes have rekindled a focus on recruiting and cultivating underrepresented students to enter higher education to mitigate the constraints of new legal changes baring the consideration of race/ethnicity in admissions.^20^

These findings demonstrate that holistic review in admissions is a useful but insufficient strategy in matriculating cohorts of students in alignment with the diversity of health care needs of our nation. Extending *holistic review* in admissions to *holistic principles* spanning developmental pathways from pre-medical education to the physician workforce is a promising next step. To compose a physician workforce for and from underserved communities, medical education must join with a diversity of partnering stakeholders to transform educational and workforce systems to (a) establish pathways from pre-K-12 education to the physician workforce, granting a greater diversity of students more and better opportunities and incentives to pursue and successfully earn an undergraduate degree in a way that qualifies them for medical school, (b) enhance holistic principles to compose and cultivate medical student cohorts increasingly for and from economically disadvantaged and marginalized communities, and (c) supporting all students and residents through innovative experiences to become practicing physicians with a profound commitment to transformative changes in health care focused on meeting and exceeding the needs of underserved communities. These kinds of transformative systems changes will benefit from models and methods guiding efforts within and across developmental stages from K-12 education to the physician workforce.^21^ This longitudinal approach can cultivate crucial social capital and nurture a deep sense of belonging. Social capital encompasses the networks and relationships that are vital for academic success that are absent for those from disadvantaged groups. In addition, this sustained model of support fosters a sense of belonging and respectful inclusion, both recognized as essential for the future of medicine. By offering a supplementary support system, it aims to create infrastructures that have been historically lacking due to systemic barriers. In the remainder of this article, we offer an ecological model to describe the status of pathways from K-12 education to the workforce, unite networks of students, families, community members, and professionals, and guide needed systems changes across four developmental stages to create a diverse medical workforce that can attend to the diverse needs of our nation.

## An Ecosystem Model of Physician Development

Bronfenbrenner^22^ established a seminal theory that evolved over time to offer a framework for understanding human development as a function of the person, context, process, and time features. Ecological systems theory has been embraced in medical education to understand and act upon people and programs to achieve mission-aligned outcomes.^23^ This theory frames pathways spanning pre-medical education, medical education, residency, and the physician workforce in terms of *person*, *context*, *process*, and *time* manifested within social networks.^24^

**FIG. 1.**
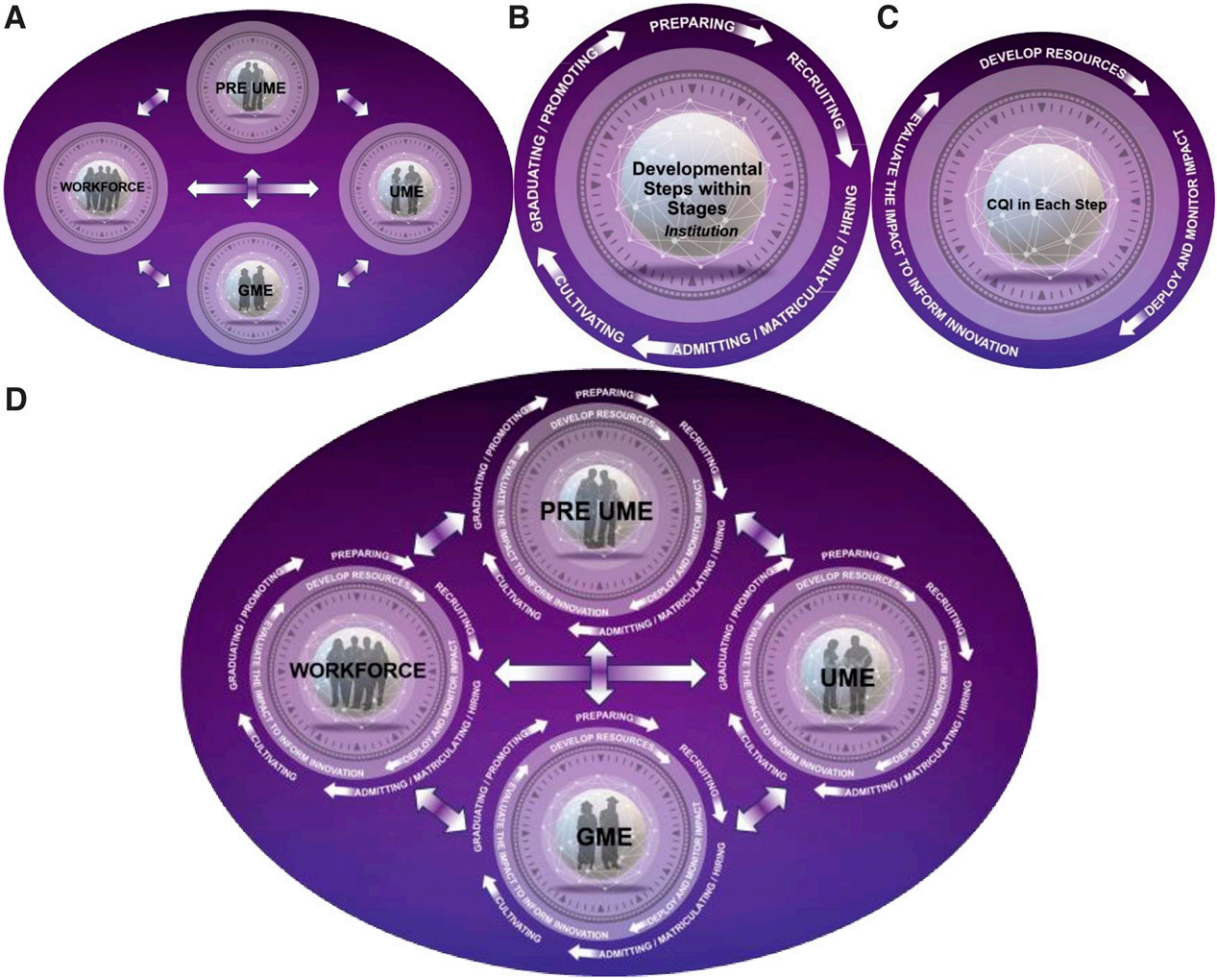
Holistic Ecosystem Model for Physician Workforce Development. Panel **(A)** Developmental Stages: Illustrates four key stages in the pathway from k-12 education to the physician workforce: Pre-Undergraduate Medical Education (PRE UME), Undergraduate Medical Education (UME), Graduate Medical Education (GME), and the Physician Workforce. PRE UME and Workforce stages span the longest durations and exert the greatest influence on identity formation and community health impact. Panel **(B)** Developmental Steps: Outlines five steps—preparation, recruitment, selection, cultivation, and transition—within each stage. These steps may occur sequentially or concurrently, shaped by available resources and networks. Holistic principles support equitable progression, especially for underrepresented and marginalized populations. Panel **(C)** Continuous Quality Improvement (CQI) process: Depicts a three-phase continuous quality improvement (CQI) cyclical process: Develop resources, deploy and monitor impact, and evaluation. This cycle enhances holistic principles across stages and steps, guiding resource creation and refinement to diversify the physician workforce and improve outcomes for underserved communities. Panel **(D)** Integrated Ecosystem Model: Combines panels, A, B, and C to yield a unified ecosystem of the pathways to the physician workforce. Encourages collaboration among stakeholders across stage and steps to align resources and support students from underserved communities. Promotes systemic change through coordinated action within institutions and networks.

These four features interact with a variety of contexts to yield developmental pathways within a human ecology. People navigate these pathways through a host of developmental processes that are often time-bound. For example, K-12 education is both a context and a developmental process that occurs within a defined period of the lifespan. The ecosystem is composed of a nested array of contexts, including the microsystem (e.g., a family, classroom, or school), mesosystem (e.g., parent-teacher association and college fairs), exosystem (e.g., the higher education system, the health care system, and the legal system), and the macrosystem (e.g., cultural elements moving certain groups toward or away from careers in health care). These different contexts and the people that inhabit them influence each other through a variety of time-bound processes. We assert that this model can be applied to a system of efforts focused upon diversifying the physician workforce and enhancing health (see Fig. 1).

We apply ecological systems theory to pathways leading to the physician workforce to yield four primary powerful developmental stages that define career pathways to the physician workforce (see Fig. 1, Panel A). The first stage is pre-undergraduate medical education, which begins at birth and proceeds for 2–3 decades of human development. Consequently, we assert that pre-medical education is the most powerful phase in moving students toward or away from a career in medicine. This phase includes family, K-12, and undergraduate and graduate educational settings that precede and prepare students for medical school admission. The second stage is undergraduate medical education, constituting four years of the lifespan. This stage determines who may become a physician and the nature of their specialty choices. The third stage is the graduate medical education period, which solidifies the specialty choice of a physician and provides the setting to hone their practices and influence their interests in the populations they will serve in the future. The final stage is the physician workforce that may continue for upwards of 70 years, as is the case for the oldest living physician at 101 years old.^24^ Their work not only influences the health of populations, they also serve as role models and educators to future generations of youth, pre-medical and medical students, and newer cohorts of physicians. While the medical education and residency phases are undoubtedly influential within the context of the overall developmental pathway, they pale in comparison to the influence of the pre-medical and workforce stages. The former two stages largely define who becomes a physician and their specialty choices. The pre-medical phase spans more than two decades and defines their identities, core values, and beliefs, and the workforce stage defines the nature and impact of their practice on the health of our population for two–four decades. These developmentally graded stages and their contexts are in constant interaction through networks of students, family members, community members, patients, educators, colleagues, and leaders spanning many contexts. These *people*, their *contexts*, the *processes* that amount to the work and interactions of these constituent groups, and *time/timing* elements all unite to yield academic pathways leading students toward or away from careers in medicine.

Within each development stage, there exists a set of general developmental steps that are advanced through a complex set of processes (see Fig. 1, Panel B). To ease the presentation, we have selected a sequence to the steps, but in practice the sequence may vary within a developmental stage as the circumstances of a student/resident/physician change. Likewise, steps can be concurrent, as in the case of a student making progress toward a degree as they are also preparing to seek admission to another degree program.

The first step within a stage involves *preparing* for the next developmental stage. In the pre-medical stage, students and their social network operate within and across settings to prepare a student for success in the medical education admissions process. In the medical school stage, students engage in clerkships to make professional connections, secure the endorsements of certain clerkship directors, and seek out certain residencies to bolster their chances of securing a desired match. Advantaged students tend to be surrounded by a rich and robust network of people across settings that prepare them for academic success and strengthen their interest in medicine. Disadvantaged students tend to lack these resources, face more barriers to academic success, and lack opportunities to develop an interest in medicine.

Underrepresented students constitute what social reproductive theory describes as “newcomers” who require greater social capital to flourish in medical school. Holistic principles in this step can be manifested in pathway programs bridging K-12, undergraduate education, medical education, residencies, and workforce settings.^26,25^ These pathways are open to all students and embrace equity principles by aligning resources to the needs of students. These pathways, for example, may offer disproportionate financial resources to students who cannot afford higher education or travel stipends to support medical students and residents to travel to residency and job interviews. More affluent students with limited experiences outside their home communities may be granted the opportunity to serve in medically underserved settings so that they can be better prepared to provide effective health care to a greater diversity of patients.

The second developmental step involves students/workers being *recruited* into the next stage. Educators in K-12 and higher education may collaborate to create programs and exposure opportunities to support students’ successful transition from K-12 to higher education and into medical education. Selective pathways programs, combined Baccalaureate/MD programs, Post-Baccalaureate/MD, and early assurance programs are examples of formalized processes to support the recruitment and progression of students into medical school. Residency directors and their associates engage in active recruitment activities through social networks to connect with desirable students who hold promise to meet the needs of residency programs. During the residency period, health systems engage in various strategies to retain and recruit residents to join their practices and especially those in need of new physicians. Employing holistic principles in this step may involve educators, recruiters, employers, and physician uniting to engage with underrepresented students and their networks in a culturally attuned partnership to grant them meaningful opportunities to become connected to educational, residency, and workplace settings that place a strong value on composing cohorts of students, residents, and physicians for and from underserved communities. More formalized efforts may include creating pathway or degree programs with an expressed focused composing a physician workforce for and from local underserved urban and rural communities to provide for their pressing health care needs.

In the third step, students/residents/workers are offered admissions, matching to a residency, or being hired. This is the *selection* phase. Students/residents/workers are selected for opportunities, and they are selecting among the offers they receive. Admissions committees at the baccalaureate or medical school levels may employ mission-aligned holistic principles and practices to evaluate and grant seat offers to prospective students. Residency directors may use these methods to evaluate and rank candidates in alignment with their mission. Employers may do the same as well. Alternatively, these parties may place a disproportionate emphasis on candidates’ characteristics that align with select priorities like academic metrics that tie to indicators of prestige, status, and power of the entities and their candidates. In doing so, they continue to contribute to the persistent disparities in educational pathways and health systems.

During the fourth developmental step, networks cultivate students/residents/workers as they cultivate themselves toward certain desired skills, statuses, and outcomes. Educators and students partner to support students in the learning process to master knowledge and skills and achieve higher academic metrics that bring a greater array of future educational and career opportunities. Families, peers, and other community members contribute human, social, and economic capital to support the cultivation process. Residency faculty and staff offer developmentally graded opportunities for residents to develop their clinical knowledge and skills to become a junior, senior, and chief resident. Employers grant their physicians professional development opportunities in the form of trainings and conferences. Employing holistic principles, educators and employers increasingly recognize the roots of disparities in hiring and progression opportunities, and they actively engage in individual, personalized, and culturally attuned efforts to support all students/residents/physicians in an equitable manner. In this case, equity is understood as a necessary aspect of equality. Equity implies customized support to grant each student/resident/physician what they need to have an equal opportunity for success.

The fifth and final step is that students/residents/physicians graduate or are promoted. They experience a role transition. Those from marginalized communities may need to devote much more effort to establishing and cultivating networks of support and securing basic resources in these transitions. These principles also help guide efforts to support graduates to have equitable opportunities to maximally benefit from their degree and to navigate transitions to the next developmental stage. Within physician careers, employers offer an array of professional development opportunities to help all employees, and especially those who are underrepresented, understand and successfully pursue career progression opportunities in a manner that reflects diversity at all physician leadership ranks. Employers actively collaborate with stakeholders in other development steps and stages to connect a diversity of physicians with a diversity of students and residents to cultivate a growing, supportive community devoted to enhancing diversity, equity, and inclusion across the entire breadth of the developmental pathway of physicians.

Holistic principles are enhanced across developmental stages and steps through a continuous quality improvement (CQI) process that includes three general phases (See Fig. 1, Panel C). In the first phase, holistic principles are employed to develop resources to advance a mission element. In the second phase, the resource is deployed. In the third phase, the resource is evaluated using varying methods commensurate with the scale of the resource and those engaged in it. The results of the third phase are employed to inform a new cycle. Employing this CQI cyclical process to create and improve resources in accord with holistic principles across developmental steps and stages contributes to the broader ecosystem devoted to medically underserved and marginalized communities by changing the composition, preparation, and goals of the physician workforce to become more for and from all communities, and especially those that are marginalized, minoritized, and underserved.

The panels in Figure 1 unite to yield an ecosystem of people, contexts, processes, and time/timing features for the physician workforce (see Fig. 1, Panel D). This ecosystem model opens the possibility of new collaborative networks because it engages stakeholders with shared aims, opportunities, challenges, and resources within and across developmental stages and steps. This model encourages, for example, admissions leaders, residency directors, and hiring managers to establish collaborative networks, and to share and align their resources derived from holistic principles to advance a shared mission. The model also encourages collaboration among educators across multiple developmental stages to collaborate and adpat their practices with a common purpose to advance students for and from underserved communities.

These kinds of collaborations may be especially powerful within large universities that include academic health centers because they have the capacity to create complete developmental pathways within one learning/working context and especially in support of underserved students who may lack the resources within their networks to successfully navigate the necessary developmental steps, stages, and their transitions. For example, these kinds of universities could (a) align their recruitment and cultivation programming with rural high school students focused on health professions with (b) health-focused baccalaureate pathways that include curriculum concentrations on rural communities, centered on underserved communities and including strong student support systems, (c) partner with their College of Medicine to prioritize these experiences and credentials in their admissions process, (d) develop a rural health pathway within their medical education curriculum to prepare future graduates to (e) enter their rural-focused residencies, (f) become rural-serving physicians, (g) eventual leaders within their health system (h) leading the nation in advancing rural health. We assert that composing a physician workforce for and from underserved communities and measurably advancing the health of these communities will require comprehensive and system change of this kind, but we offer that these large-scale changes will likely spring from a collection of smaller efforts rooted in existing resources and aligned with an ecosystem model.

Comprehensive pathways to the physician workforce could be especially beneficial to states and communities suffering from the looming high school enrollment cliff, outflows of residents to other states, challenges in recruiting and retaining physicians, and significant educational and health disparities. The ecosystem model spurs significant changes in how we think about the developmental pathways to the physician workforce and how we may mobilize resources to promote progress and ease transitions and especially for underrepresented students who face many fewer opportunities and many more challenges along their journey. More broadly, the ecosystem model challenges academic medicine to take stock of its vast resources and networks to cultivate robust pathways from and to underserved communities to help enhance their health, education, and economic opportunities.

## Abbreviations Used

AAMC: Association of American Medical Colleges
CQI: Continuous quality improvement
GME: Graduate medical education
OECD: Organization for Economic Co-operation and Development
PRE UME: Pre-undergraduate medical education
UME: Undergraduate medical education
